# Development and Validation of a Novel Indicator of Visual Disability in the National Health and Aging Trends Study

**DOI:** 10.1093/geroni/igab018

**Published:** 2021-06-10

**Authors:** Lindsey B De Lott, Ajay Kolli, Yunshu Zhou, Mengyao Hu, Joshua R Ehrlich

**Affiliations:** 1 Department of Ophthalmology & Visual Sciences, Michigan Medicine, University of Michigan, Ann Arbor, USA; 2 Harvard T.H. Chan School of Public Health, Harvard University, Boston, Massachusetts, USA; 3 Institute for Social Research, University of Michigan, Ann Arbor, USA

**Keywords:** Aging, NHATS, Psychometrics, Vision

## Abstract

**Background and Objectives:**

The National Health and Aging Trends Study (NHATS) is an ongoing, nationally representative panel study of older adults that collects data on health and disability, including measures on self-reported visual disability (SRVD). Prior studies assessing the association of SRVD with other measures of healthy aging have classified participants as having or not having SRVD, which does not capture the full spectrum of SRVD reported by participants. Therefore, we sought to develop and validate an ordinal indicator of SRVD to facilitate research on the impact of late-life SRVD on health and disability in NHATS.

**Research Design and Methods:**

We used 2015 NHATS data with community-dwelling participants who answered survey questions about visual functioning and vision aid use. Based on responses, participants were categorized into one of 6 groups: blind, near and distance SRVD without vision aid use, near and distance SRVD with vision aid use, near or distance SRVD without vision aid use, near or distance SRVD with vision aid use, or no SRVD. Multivariable Poisson regression models assessed convergent validity of the ordinal SRVD scale with functional activity and well-being scores, while adjusting for demographic factors and medical comorbidities.

**Results:**

Of the 7061 eligible individuals, 8.3% (*n* = 742) reported SRVD. Using our novel ordinal indicator of SRVD in NHATS, higher levels of SRVD were significantly associated with lower functional activity scores (*p* < .001 for all) and subjective well-being (*p* < .001), except for participants reporting blindness. Significant differences between SRVD groups were found, which could not be captured using a binary SRVD variable.

**Discussion and Implications:**

A novel 6-level SRVD scale in NHATS demonstrated convergent validity with functional activity and well-being scales. This scale provides a new tool with improved measurement precision to study the impact of late-life SRVD on health and disability in a nationally representative study of older adults.

**Translational Significance:** We developed a new, more nuanced variable for assessing the impact of vision loss on older adults in the United States participating in the National Health and Aging Trends Study (NHATS). We demonstrated the validity of this variable, observed significant differences among people with different levels of vision loss, and found that it explained a greater proportion of the variance in key gerontological outcomes than existing indicators of vision impairment in NHATS. Findings will facilitate future research on the impact of self-reported visual disability on health and disability in older adults.

## Background and Objectives

Vision loss is common among older adults, affecting 1 in 11 people older than age 65 in the United States and with those older than the age of 80 at the greatest risk of blindness (1). Vision loss among older adults is associated with increased falls (2,3), fear of falling (3–5), reduced activity and mobility (6), decreased well-being (7,8), and even mortality (9). Consequently, understanding the complex relationship between vision loss, physical disability, and well-being is important for designing programs and policies that promote healthy aging.

Typically, epidemiologic studies have used one of the 3 different measures to assess vision and its impact on the lives of older adults. First, vision can be assessed with objective vision tests (eg, visual acuity). Second, vision can be measured with survey responses of vision quality (eg, good to poor, self-reported visual disability [SRVD]). Third, vision can be assessed using survey questions about the impact of vision on specific activities and tasks (eg, vision-dependent functioning). Although objective vision tests provide quantitative measures of vision loss, how older adults perceive their own visual function may differ from quantitative measurements (10). SRVD has been independently associated with fear of falling and fear-related activity restriction even when visual acuity and contrast sensitivity were not (4). Similarly, tasks, such as watching television or reading a book, have been associated with a person’s satisfaction with their vision (11,12). Therefore, SRVD measures a distinct and valuable construct that may have important influences on physical disability and well-being in older adults.

The National Health and Aging Trends Study (NHATS) is a nationally representative panel study of Medicare beneficiaries in the United States aged 65 years and older. It provides a unique platform for the study of late-life disability trends and trajectories. Prior research on SRVD in NHATS has relied on a dichotomous indicator of poor vision, which may lack the measurement precision needed to study the impact of SRVD trajectories on health, disability, and well-being. The purpose of this study was to develop and validate a novel ordinal indicator of SRVD in NHATS using existing survey questions with binary response options. We anticipate that this variable would facilitate future studies on the impact of SRVD on late-life health and disability.

## Method

### Data Source and Study Population

The baseline NHATS sample was recruited in 2011 from the Medicare enrollment file and replenished in 2015 due to loss of follow-up and death (13). The oldest age groups and non-Hispanic Black older adults were oversampled. After informed consent, NHATS conducts in-person interviews annually, including detailed assessments of disability, well-being, and assessment of SRVD (14). The analytic sample (*n* = 7061) for this study included all community-dwelling NHATS participants from the replenished 2015 cohort with either complete data for all of the variables in our study or imputed values provided by NHATS when response data were not available. Applying this approach, data were available for over 94% of participants. This study was deemed exempt by the University of Michigan Institutional Research Board because it consisted of publically available deidentified data.

### Functional Ability and Subjective Well-Being Scores

Validated NHATS scales were used to predict functional ability (household, self-care, mobility activities) and subjective well-being scales (15). Three types of functional ability were assessed, including household activities (constructed using 5 items), self-care activities (constructed using 4 items), and mobility activities (constructed using 3 items). For each activity, a validated 4-level measure (16) was used to classify performance while accounting for behavioral and environmental adaptations. The range of scores is from 5 to 20 for household activities, 4–16 for self-care, and 3–12 for mobility with higher scores representing more independent functional ability. Subjective well-being was constructed using a validated 7-item indicator: 4 items that reflect positive and negative emotions (frequency of feeling cheerful, bored, full of life, or upset in the last month) and 3 items that reflect self-realization (extent of disagreement with statements about purpose in life, self-acceptance, and environmental mastery). The well-being score ranges from 0 to 22, with a higher score indicating better well-being. The scale has good internal consistency and factor loadings that indicate a single factor model (15,17). Subjective well-being was not reported for participants requiring a proxy.

### Self-Reported Visual Disability

To assess SRVD, participants were asked multiple yes/no questions to assess their vision. They are first asked if they have difficulty seeing at a distance. Participants can respond yes, no, or blind. Participants are then asked to consider distance tasks: whether they see well enough when wearing their glasses or contact lenses: (a) to recognize someone across the street, and if they respond “yes,” whether they also see well enough to (b) view the television across the room. Similarly, participants are asked about the use of glasses or contact lenses to see things close up and whether they still see well enough to read newspaper print. Last, they are asked 2 questions about vision aids (VAs) to help see things close up or read newspaper print. VAs are defined by NHATS as including “things like magnifying glass[es], large print books, and other tools” to help people see. Because the use of the term “vision aid” within NHATS could be interpreted by a participant as a low-vision device (eg, magnifiers) or corrective lenses (eg, reading glasses), we considered any participant using glasses, contact lenses, or an NHATS-defined low VA as using a VA.

Participants were classified as “blind” based on self-reported blindness. For the remaining participants, 2 authors with eye care expertise (L.B.D. and J.R.E.) assessed responses to questions about near and distance vision with and without VA use. A 6-level variable was associated with scores on the functional activity and well-being scales in an ordered fashion. While the top level, “blind” did not consistently follow the ordered pattern, based on its conceptual meaning it was retained as the most extreme level of the scale. In the final ordinal scale, participants were categorized into one of the 6 groups: blind, near and distance SRVD without VA use, near and distance SRVD with VA use, near or distance SRVD without VA use, near or distance SRVD with VA use, or no SRVD regardless of VA use. By comparison, prior studies have employed a binary definition of SRVD if the participant reported blindness or difficulty reading or recognizing someone across the street when wearing corrective lenses (3,18,19).

### Other Covariates

Other covariates include age, sex, race, ethnicity, educational level, chronic medical comorbidities, income, and use of a proxy to answer survey questions, which were collected for all participants. We used a count of chronic conditions to assess medical comorbidities (14): history of a heart attack, heart disease, high blood pressure, arthritis, osteoporosis, diabetes, lung disease, stroke, Alzheimer’s disease or dementia, cancer (excluding skin), a broken or fractured hip, and current symptoms of depression from the Patient Health Questionnaire-2 (20,21).

### Statistical Analyses

The sample was characterized using survey-weighted means for continuous variables and frequencies for categorical variables. Differences in participant characteristics by SRVD were assessed using Pearson chi-squared test for categorical variables and one-way analysis of variance for continuous variables.

Generalized linear regression models with a Poisson distribution and log-link function were used to test associations between SRVD, measured by the binary and ordinal scale, and outcomes (functional ability and well-being scores). Both unadjusted and adjusted models (ie, controlling for other covariates) were performed with an examination of *R*^2^ values and residual plots for each model. The models demonstrated a good fit of the data without violations of model assumptions. All tests were two-sided, and a *p* value of less than .05 was considered statistically significant. All models accounted for the complex NHATS survey design, including sampling units, strata, and weights. All analyses were performed using SAS version 9.4 (SAS Institute, Cary, NC) and Stata Statistical Software: Release 16 (StataCorp 2019; StataCorp LLC, College Station, TX).

## Results

Distributions of the binary and ordinal visual scales, overall, by demographic characteristics and proxy status, are presented in Table 1. Overall, 91.7% of participants did not have SRVD. Based on the 6-category ordinal scale, the 8.3% with SRVD were further categorized: 2.5% had near or distance SRVD and used VA, 3.6% had near or distance SRVD without VA, 0.7% had near and distance SRVD with VA, 1.1% had near and distance SRVD without VA, and 0.4% were blind.

**Table 1.** Weighted Sample Characteristics by Self-Reported Visual Disability Status and Scale Type

Characteristic*	2-Level Scale			6-Level Scale						
	No SRVD	Any SRVD†	*p* ‡	No SRVD	Near or distance SRVD with vision aids	Near or distance SRVD without vision aids	Near and distance SRVD with vision aids	Near and distance SRVD without vision aids	Blind	*p* ‡
Total, %	91.7	8.3		91.7	2.5	3.6	0.7	1.1	0.4	
Age, years, %			<.0001							<.0001
65–69	30.2	22.1		30.2	25.6§	25.9	8.7§	9.9§	20.4§	
70–74	27.3	20.5		27.3	22.4	24.7	8.3§	15.3§	5.3§	
75–79	18.8	17.3		18.8	16.5	18.0	20.2§	16.6§	13.8§	
80–84	12.6	13.4		12.6	13.0	11.8	18.3§	15.4§	16.5§	
85–89	7.5	14.5		7.5	15.2	9.6	22.7§	21.7	20.8§	
90+	3.6	12.2		3.6	7.3§	10.0	21.9§	21.1	23.2§	
Sex, %			.0002							.0006
Male	45.3	35.2		45.3	29.1	39.6	29.4§	36.8	38.4§	
Female	54.7	64.8		54.7	70.9	60.4	70.6	63.2	61.6§	
Race/ethnicity, %			<.0001							<.0001
White, non-Hispanic	82.0	68.2		82.0	80.0	60.1	86.8	57.4	65.8§	
Black, non-Hispanic	8.1	11.9		8.1	6.3	16.7	5.2§	13.7	10.6§	
Other, non-Hispanic	3.5	4.9§		3.5	4.0§	4.8§	4.8§	6.4§	7.9§	
Hispanic	6.4	15.0		6.4	9.7§	18.5	3.2§	22.4§	15.6§	
Income quartile, %			<.0001							<.0001
<$17 000	17.6	39.1		17.6	30.0	39.8	32.8§	57.3	49.7§	
$17 000–$30 999	21.3	25.3		21.3	29.4	23.4	29.5§	16.8§	34.2§	
$31 000–$59 999	26.2	21.7		26.2	21.9	23.3	25.6§	18.4§	8.2§	
≥$60 000	34.9	13.9		34.9	18.7	13.5	12.1§	7.5	7.9§	
Chronic conditions‖ (0–13), mean (*SD*)	2.58 (0.03)	3.73 (0.11)	<.0001	2.58 (0.03)	3.75 (0.15)	3.46 (0.18)	4.08 (0.32)	4.27 (0.32)	3.93 (0.33)	<.0001
Education			<.0001							<.0001
Less than high school	15.3	34.8		15.3	26.4	39.2	30.6§	45.1	25.8§	
High school	25.8	29.3		25.8	29.0	27.9	34.4§	29.4§	34.9§	
Some college	23.7	17.8		23.7	22.4	15.7	14.6§	14.5§	22.5§	
College degree	35.3	18.1		35.3	22.2	17.2	20.4§	10.9§	16.8§	
Proxy	2.4	15.4	<.0001	2.4	10.6	13.4	10.3	38.1	10.0	<.0001
*N*	6319	742		6319	198	325	62	113	44	

*Note:* SRVD = self-reported visual disability.

*Data are presented as weighted proportions of US population of Medicare beneficiaries aged 65 or older, accounting for the study design of the National Health and Aging Trends Study.

^†^Includes participants who self-reported near and/or distance vision impairment.

^‡^*p* values are unadjusted and calculated using design-adjusted Pearson chi-squared test for categorical variables and *t*-tests for continuous variables.

^§^The unweighted count is less than 30; therefore, the weighted percentages may not be reliable.

^‖^Count of chronic conditions to reflect multimorbidity: history of a heart attack, heart disease, high blood pressure, arthritis, osteoporosis, diabetes, lung disease, stroke, Alzheimer’s disease or dementia, cancer (excluding skin), a broken or fractured hip, and current symptoms of depression (from the validated Patient Health Questionnaire-2).

Distributions of both binary and ordinal vision scales varied by demographic characteristics and proxy status. Among those with no SRVD, 3.6% were older than 90 years. The proportion of participants older than 90 increases across the SRVD groups, up to 23.2% among those who are blind. By gender, 54.7% of those with no SRVD were female, while 64.8% reporting any SRVD were female. Compared to those with no SRVD, those with any SRVD contain higher proportions of non-Hispanic Black and Hispanic participants. Those participants with any SRVD had lower education, lower incomes, more chronic conditions, and were more likely to have a proxy respondent, compared to those with no SRVD. In general, similar patterns have been observed for the 6-level SRVD scale in terms of distributions in these demographic and socioeconomic characteristics.

Table 2 presents the weighted mean scores for functional ability and subjective well-being scores by the binary and ordinal SRVD scales. For the binary scale, participants with no SRVD had higher weighted mean functional ability and well-being scores. Specifically, the mean scores (95% CI) for mobility, self-care, and household activities for those with any SRVD were 8.3 (8.0–8.5), 12.0 (11.6–12.3), and 13.6 (13.1–14.1), respectively, while the values were 10.6 (10.6–10.7), 14.3 (14.3–14.4), and 17.9 (17.7–18.0), respectively, for those without SRVD. For subjective well-being, those with SRVD had a mean of 15.6 (15.1–16.1) and those without SRVD had a mean of 17.5 (17.4–17.6).

**Table 2.** Weighted Mean Scores for Activity (mobility, self-care, and household activity) and Well-Being by the 2- and 6-Level Self-Reported Visual Disability Scales

SRVD Scale Type	Summary Across Activities		Mean Activity Scores			Mean Well-Being Score (95% CI)
	% (95% CI)	No. (in millions)	Mobility (95% CI)	Self-care (95% CI)	Household activity (95% CI)	
*2-level scale*						
No SRVD	91.7 (90.8–92.5)	34.9	10.6 (10.6–10.7)	14.3 (14.3–14.4)	17.9 (17.7–18.0)	17.5 (17.4–17.6)
SRVD*	8.3 (7.5–9.2)	3.2	8.3 (8.0–8.5)	12.0 (11.6–12.3)	13.6 (13.1–14.1)	15.6 (15.1–16.1)
*6-level scale*						
No SRVD	91.7 (90.8–92.5)	34.9	10.6 (10.6–10.7)	14.3 (14.3–14.4)	17.9 (17.7–18.0)	17.5 (17.4–17.6)
Near or distance SRVD with vision aids	2.5 (2.0–3.0)	0.97	8.9 (8.4–9.5)	12.9 (12.3–13.4)	15.3 (14.5–16.1)	15.8 (15.1–16.5)
Near or distance SRVD without vision aids	3.6 (3.1–4.2)	1.4	8.6 (8.2–9.1)	12.4 (11.9–12.9)	14.2 (13.5–14.9)	15.7 (14.8–16.6)
Near and distance SRVD with vision aids	0.7 (0.5–0.8)	0.25	7.5 (6.8–8.3)	11.5 (10.6–12.5)	12.1 (10.5–13.6)	15.0 (14.1–15.9)
Near and distance SRVD without vision aids	1.1 (0.8–1.4)	0.42	6.4 (5.6–7.2)	9.2 (8.2–10.1)	10.3 (9.0–11.6)	14.7 (13.4–16.1)
Blind	0.4 (0.3–0.6)	0.15	7.4 (6.1–8.8)	10.3 (8.4–12.2)	9.4 (7.5–11.4)	16.3 (15.1–17.6)

*Note:* SRVD = self-reported visual disability; CI = confidence interval.

*Includes participants who self-reported near and/or distance SRVD

**Figure 1.**
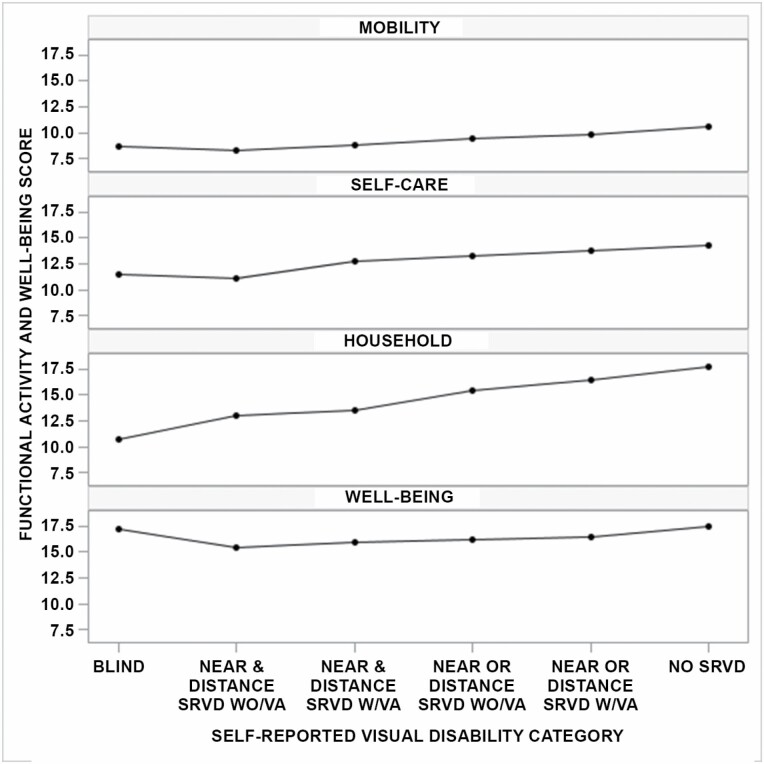
Mean-adjusted functional activity and well-being scores by self-reported visual disability category. SRVD WO/VA = self-reported visual disability without vision aid; SRVD W/VA = self-reported visual disability with vision aid.

For the 6-level ordinal scale, functional activity and well-being scores trended lower as SRVD worsened in dose–response fashion. For those participants who reported blindness, the mean (95% CI) mobility and self-care scores were higher (7.4 [6.1–8.8] and 10.3 [8.4–12.2], respectively) than the preceding category—near and distance SRVD without VA (6.4 [5.6–7.2] and 9.2 [8.2–10.1], respectively). For well-being, the mean score (95% CI) for participants reporting blindness was 16.3 (15.1–17.6), which was higher than for other SRVD categories. For household activities, scores decreased progressively from no SRVD to blindness, a trend that was largely observed across the other outcomes. Figure 1 shows the mean-adjusted functional activity and well-being scores by the SRVD category.

As given in Table 3, the regression coefficients indicated worse functional activity and subjective well-being among those with greater SRVD on the ordinal scale compared to those with less SRVD, even after controlling for participants’ health, demographic variables, and proxy status. As above, those with blindness paradoxically had better functional activity and well-being scores except for household activities. Significant differences between those with different levels of SRVD were also observed (Supplementary Table 1). Table 4 provides the adjusted predicted functional activity and well-being scores.

**Table 3.** Weighted Unadjusted and Adjusted Models of Functional Ability and Well-Being Scores by Level of Self-Reported Visual Disability Using Poisson Regression

Variable	Mobility Score		Self-Care Score		Household Activity Score		Well-Being Score	
	β (95% CI)	*p*	β (95% CI)	*p*	β (95% CI)	*p*	β (95% CI)	*p*
*Unadjusted models*								
Level of SRVD (ref = No SRVD)								
Near or distance SRVD with vision aids	−0.17 (−0.23, −0.11)	<.001	−0.11 (−0.15, −0.06)	<.001	−0.16 (−0.21, −0.10)	<.001	−0.10 (−0.15, −0.06)	<.001
Near or distance SRVD without vision aids	−0.21 (−0.26, −0.16)	<.001	−0.15 (−0.19, −0.10)	<.001	−0.23 (−0.28, −0.18)	<.001	−0.11 (−0.16, −0.05)	<.001
Near and distance SRVD with vision aids	−0.34 (−0.44, −0.25)	<.001	−0.22 (−0.30, −0.14)	<.001	−0.39 (−0.52, −0.26)	<.001	−0.15 (−0.22, −0.09)	<.001
Near and distance SRVD without vision aids	−0.51 (−0.62, −0.39)	<.001	−0.45 (−0.55, −0.34)	<.001	−0.55 (−0.67, −0.42)	<.001	−0.16 (−0.25, −0.07)	.001
Blind	−0.36 (−0.54, −0.17)	<.001	−0.33 (−0.52, −0.14)	.001	−0.64 (−0.84, −0.43)	<.001	−0.07 (−0.14, 0.01)	.076
*Adjusted models*								
Level of vision impairment (ref = No SRVD)								
Near or distance SRVD with vision aids	−0.07 (−0.12, −0.03)	.002	−0.03 (−0.06, −0.00)	.046	−0.07 (−0.11, −0.03)	.002	−0.05 (−0.10, −0.01)	.012
Near or distance SRVD without vision aids	−0.10 (−0.14, −0.07)	<.001	−0.07 (−0.11, −0.04)	<.001	−0.14 (−0.18, −0.09)	<.001	−0.08 (−0.13, −0.03)	.002
Near and distance SRVD with vision aids	−0.19 (−0.28, −0.10)	<.001	−0.11 (−0.19, −0.03)	.010	−0.27 (−0.39, −0.14)	<.001	−0.09 (−0.15, −0.02)	.010
Near and distance SRVD without vision aids	−0.25 (−0.34, −0.16)	<.001	−0.25 (−0.34, −0.16)	<.001	−0.31 (−0.41, −0.20)	<.001	−0.12 (−0.19, −0.05)	.002
Blind	−0.19 (−0.37, −0.01)	.040	−0.22 (−0.41, −0.03)	.025	−0.50 (−0.70, −0.30)	<.001	−0.01 (−0.09, 0.06)	.709
Age, years (ref = 65–69)								
70–74	0.00 (−0.01, 0.02)	.965	−0.00 (−0.02, 0.01)	.433	0.01 (−0.00, 0.02)	.099	0.02 (0.00, 0.04)	.020
75–79	−0.00 (−0.01, 0.01)	.982	−0.01 (−0.02, 0.00)	.188	0.02 (−0.00, 0.03)	.055	0.03 (0.01, 0.05)	.001
80–84	−0.04 (−0.06, −0.02)	<.001	−0.04 (−0.05, −0.02)	<.001	−0.02 (−0.03, 0.00)	.056	0.03 (0.01, 0.05)	.001
85–89	−0.07 (−0.10, −0.05)	<.001	−0.06 (−0.08, −0.04)	<.001	−0.05 (−0.08, −0.03)	<.001	0.03 (0.01, 0.05)	.007
90+	−0.20 (−0.23, −0.17)	<.001	−0.14 (−0.16, −0.12)	<.001	−0.14 (−0.18, −0.10)	<.001	0.03 (0.00, 0.05)	.045
Sex (ref = male)								
Female	−0.02 (−0.03, −0.01)	.004	−0.01 (−0.02, −0.00)	.003	−0.03 (−0.04, −0.01)	<.001	0.00 (−0.01, 0.01)	.941
Race/ethnicity (ref = White, non-Hispanic)								
Black, non-Hispanic	−0.02 (−0.04, −0.00)	.022	0.00 (−0.01, 0.02)	.474	−0.03 (−0.05, −0.01)	.001	0.04 (0.03, 0.05)	<.001
Other, non-Hispanic	−0.02 (−0.06, 0.02)	.250	0.01 (−0.03, 0.04)	.723	−0.05 (−0.08, −0.02)	.004	0.00 (−0.04, 0.05)	.896
Hispanic	−0.01 (−0.04, 0.02)	.542	0.01 (−0.01, 0.03)	.241	0.00 (−0.03, 0.02)	.550	0.06 (0.04, 0.08)	<.001
Education (ref = college)								
Less than high school	−0.00 (−0.02, 0.02)	.885	0.01 (−0.01, 0.03)	.306	−0.02 (−0.04, −0.00)	.019	−0.00 (−0.02, 0.02)	.888
High school	0.01 (−0.01, 0.02)	.227	0.00 (−0.01, 0.01)	.571	−0.01 (−0.02, 0.01)	.453	−0.01 (−0.02, 0.01)	.358
Some college	−0.00 (−0.01, 0.01)	.954	−0.00 (−0.01, 0.01)	.761	0.00 (−0.01, 0.01)	.536	−0.00 (−0.02, 0.01)	.883
Income quartile, % (95% CI) (ref ≥$60 000)								
<$17 000	−0.07 (−0.09, −0.05)	<.001	−0.03 (−0.05, −0.02)	<.001	−0.05 (−0.08, −0.03)	<.001	−0.08 (−0.10, −0.06)	<.001
$17 000–$30 999	−0.03 (−0.05, −0.01)	.001	−0.02 (−0.03, −0.01)	.003	−0.02 (−0.04, −0.01)	.002	−0.05 (−0.07, −0.04)	<.001
$31 000–$59 999	0.00 (−0.01, 0.01)	.947	−0.01 (−0.01, 0.00)	.194	0.00 (−0.01, 0.01)	.757	−0.02 (−0.03, −0.01)	.002
Chronic conditions (0–13), Mean (*SD*)	−0.05 (−0.05, −0.04)	<.001	−0.03 (−0.03, −0.03)	<.001	−0.03 (−0.03, −0.02)	<.001	−0.04 (−0.04, −0.03)	<.001
Proxy* (ref = no)								
Yes	−0.37 (−0.45, −0.30)	<.001	−0.36 (−0.43, −0.29)	<.001	−0.43 (−0.50, −0.36)	<.001	—	

*Note:* SRVD = self-reported visual disability; CI = confidence interval.

*All proxy-assessed surveys are missing well-being scores.

**Table 4.** Expected Functional Ability and Well-Being Scores*

Level of SRVD	Mobility Score (95% CI)	Self-Care Score (95% CI)	Household Activity Score (95% CI)	Well-Being Score (95% CI)
No vision impairment	10.5 (10.5–10.6)	14.2 (14.2–14.3)	17.7 (17.6–17.8)	17.4 (17.3–17.5)
Near or distance SRVD with vision aids	9.8 (9.4–10.2)	13.8 (13.4–14.2)	16.5 (15.8–17.2)	16.5 (15.8–17.2)
Near or distance SRVD without vision aids	9.5 (9.1–9.8)	13.2 (12.8–13.7)	15.4 (14.7–16.1)	16.1 (15.4–16.9)
Near and distance SRVD with vision aids	8.7 (8.0–9.5)	12.8 (11.8–13.8)	13.6 (11.9–15.2)	16.0 (14.9–17.0)
Near and distance SRVD without vision aids	8.2 (7.5–9.0)	11.1 (10.1–12.1)	13.0 (11.6–14.4)	15.5 (14.3–16.6)
Blind	8.7 (7.1–10.3)	11.5 (9.3–13.6)	10.7 (8.6–12.8)	17.2 (16.0–18.4)

*Note:* SRVD = self-reported visual disability; CI = confidence interval.

*Adjusted for age, sex, race/ethnicity, education, income, chronic conditions, and proxy status.

To evaluate model fit, we compared adjusted *R*^2^ between models with SRVD as a dichotomous variable (eg, 2-level scale) or SRVD as an ordinal variable (eg, 6-level scale; Table 5). For each functional activity outcome and the well-being outcome, the model using SRVD as a 6-level categorical variable resulted in a higher *R*^2^ compared to the model with a dichotomous SRVD variable, suggesting that the 6-level SRVD scale explains more variance in functional activity and well-being scores compared to the binary variable.

**Table 5.** Adjusted *R*^2^ for Models Predicting Activities and Well-Being Scores Using Binary Versus 6-Level Self-Reported Visual Disability Scale

Predicator	Outcome			
	Mobility	Self-care	Household activity	Well-being score
Binary SRVD scale	0.3299	0.3280	0.2971	0.1512
Six-level SRVD scale	0.3317	0.3342	0.3054	0.1520

*Note:* SRVD = self-reported visual disability.

## Discussion and Implications

We have developed and validated a new ordinal indicator of SRVD based on the series of self-reported vision questions collected in NHATS. We found that 8.3% of adults older than 65 years in the NHATs cohort report some degree of SRVD and 0.44% report blindness. In models adjusted for sociodemographic and medical comorbidities, worse SRVD was significantly associated with lower functional activity scores and subjective well-being. We also found significant differences between SRVD groups, which cannot be captured in the commonly used binary SRVD versus no SRVD variable. In comparison to a binary scale of SRVD versus no SRVD, our 6-level scale explained slightly more of the variance in functional and subjective well-being outcomes. The 6-level ordinal scale appears to provide a more precise measure of SRVD compared to the 2-level scale and therefore may be a useful new instrument for assessing the impact of SRVD severity on the health, disability, and well-being of older adults.

The association of worse functional activity and subjective well-being with higher levels of SRVD confirms the predictive validity of our scale. However, in participants who reported blindness, there was a paradoxical finding of better mobility, self-care, and subjective well-being compared to those with lower levels of SRVD. A similar pattern was seen in a cross-sectional evaluation that applied the World Health Organization International Classification of Functioning, Disability, and Health to participants with vision loss measured quantitatively (22). Compared to those with moderate to severe vision loss, individuals with blindness were less likely to report activity restrictions in various domains, including walking, carrying objects, fine motor skills, acquiring new skills, toileting, and reading, among others (22). It is not immediately clear why blind participants in our study and others report better physical functioning, but one possibility is that blind participants may be more likely to have longstanding causes of vision loss and to have adapted over time. Additionally, although poorer vision is generally associated with greater functional impairment (23,24), it is possible that enhanced care or low VAs and vision rehabilitative services could be more readily accessed by those with blindness, leading to a subsequent restoration of functional status. Last, this finding may reflect that blindness was assessed by asking a question about the participant’s ability to see, not function or engage in specific tasks.

A similar trend among older adults with blindness has been reported for subjective well-being in prior studies. In a qualitative study on individuals with vision loss due to diabetic retinopathy, participants reported that vision loss that is partial, deteriorating gradually, or fluctuating caused more psychological distress than total blindness (25). The authors suggested that transitioning from partial vision loss to full blindness might facilitate transition into the healing phase of a grief reaction. Similar to our study, increased anxiety, depression, and psychosocial distress have been measured in adults with vision loss. However, in many studies, a dichotomous variable is used to define vision status, so those with blindness and other levels of vision impairment may be categorized together (26,27). Notwithstanding, a higher risk of depression has been reported in individuals with blindness compared to those with and without vision loss (28). Taken together, there is an urgent need to understand the relationship between vision loss, blindness, and subjective well-being.

Estimates of the prevalence of self-reported vision loss and blindness in adults older than 65 in the United States vary widely. However, the weighted mean prevalence of blindness across 5 national surveys was 4.2% in adults aged 65–84 and 12.5% in adults aged 85 or older (29). Across the same surveys, the weighted mean prevalence of SRVD was 8.6% in adults aged 65–84 and 15. 0% in adults aged 85 or older (29). By comparison, the prevalence of SRVD among NHATS participants was similar (7.9%) and increased with age, but estimates of blindness were lower. Differences in blindness estimates are likely related to the varied questions used to assess self-reports of vision function across surveys. Future rounds of NHATS will include objective measures of visual acuity and contrast sensitivity (30), which will permit investigations on the impact of self-reported and objectively assessed vision on health, disability, and well-being outcomes.

About half of participants who reported impaired vision-dependent functioning did not use VAs; the reasons for this are likely multifactorial. Diseases such as diabetic retinopathy, glaucoma, and age-related macular degeneration are common among older adults and cause vision loss that is not correctable with glasses. Similarly, these same diseases disproportionately affect minority populations in the United States (31–33), which may explain, in part, the increased prevalence of vision loss among non-Hispanic Black and Hispanic older adults. Furthermore, the NHATS cohort is recruited from Medicare, which does not routinely pay for corrective lenses or low VAs. In a nationally representative study of older adults, those from racial and ethnic minority groups were significantly less likely to use low VAs, such as magnifiers (28). Provision of basic eye screening examinations, educating aging adults about the utility of eye exams to detect asymptomatic and treatable eye disease, and provision of corrective lenses have the potential to affect late-life functional activity and well-being.

Our study has important limitations. Participants who required the use of a proxy were excluded from analyses of well-being. This is important as proxy use was at least 10% among participants with SRVD, 2.4% among those without SRVD, and highest among those with near and distance SRVD without the use of VAs (38%). Additionally, self-reported data are susceptible to reporting biases. We also did not determine if the limitations in functional activities and well-being are due to SRVD directly or other medical and social conditions. Furthermore, the differences in functional activity and well-being scores are small between the different categories of SRVD. Although these differences between SRVD categories are significant across the studied population, it is unclear if these differences are clinically significant for individual participants. This study also had a number of strengths. Because data are nationally representative, our findings are generalizable to a large population that is at high risk for visual and functional disability. In validating the new 6-level SRVD variable, we considered multiple complementary outcomes, which contributes to our confidence in the utility of the scale.

We have developed a new ordinal SRVD scale based on survey questions in NHATS. As the importance of good visual function for maintenance of health, well-being, economic security, and independence in older adults is increasingly recognized (34), it is important to develop measures to enable the study of the impact of SRVD severity on late-life health and disability. The new scale we have developed may be useful in assessing the impact of changes in SRVD over time and the effect of vision restoring or vision preserving therapies on physical disability and well-being in NHATS.

## Supplementary Material

igab018_suppl_Supplementary_Materials
