# Supplementary material for: Development and Validation of a Novel Indicator of Visual Disability in the National Health and Aging Trends Study
Source: Innov Aging. 2021 Jun 10;5(2):igab018. doi: 10.1093/geroni/igab018 (PMC8374875; doi:10.1093/geroni/igab018)
Supplement: igab018_suppl_Supplementary_Materials [file igab018_suppl_supplementary_materials.docx]

Supplemental Table 1. Comparisons between levels of self-reported visual disability.

| Pairwise SRVD level comparison | Blind | Near and distance SRVD without vision aids | Near and distance SRVD with vision aids | Near or distance SRVD without vision aids | Near or distance SRVD with vision aids |
| --- | --- | --- | --- | --- | --- |
| No Vision Impairment | X ^a, b, c^ | X ^a, b, c, d^ | X ^a, b, c, d^ | X ^a, b, c, d^ | X ^a, b, c, d^ |
| Near or distance SRVD with vision aids | X ^c^ | X ^a, b, c^ | X ^a, c^ | X ^c^ |  |
| Near or distance SRVD without vision aids | X ^c^ | X ^a, b, c^ |  |  |  |
| Near and distance SRVD with vision aids | X ^c^ | X ^b^ |  |  |  |
| Near and distance SRVD without vision aids | X ^d^ |  |  |  |  |

Abbreviation: SRVD, self-reported visual disability.

X = significant (p < .05) in at least one model for each outcome.

^a^ significant difference in mobility model

^b^ significant difference in self-care activity model

^c^ significant difference in household activity model

^d^ significant difference in well-being model
